# Considerations for the Use of Semi-Transparent Metallic Thin Films as Potential Transmittance Standards in Spectrophotometry*

**DOI:** 10.6028/jres.080A.063

**Published:** 1976-08-01

**Authors:** R. Mavrodineanu

**Affiliations:** Institute for Materials Research, National Bureau of Standards, Washington, D.C. 20234

**Keywords:** Evaporated metal-on-quartz, filters, transmittance, neutral filters, standard reference materials, transmittance characteristics, ultraviolet-visible filters

## Abstract

Various characteristics of evaporated metal-on-fused silica filters are discussed in relation to their optical transmission properties. Special metal holders provided with shutters were designed to be used with these filters, and are described in detail. Transmittance measurements, performed in various conditions, are reported and indicate that the evaporated metal-on-fused silica filters might present an acceptable material as transfer standards in spectrophotometry.

The use of solid materials, in the form of glass filters and of solutions of inorganic and sometime organic compounds, to test the correct functioning of spectrophotometers is a common practice [1].^1^ A variety of materials are available from the National Bureau of Standards (NBS) which can be used as transfer standards to verify the accuracy of the transmittance scale and the short and long term stabilities of conventional spectrophotometers. Two Standard Reference Materials (SRM’s) have been issued by NBS for the verification of the accuracy of the transmittance scale; these are SRM 930 and SRM 931.

SRM 930 is a solid material which consists of a set of three glass filters having nominal transmittance of 10; 20; and 30 percent. They are certified for transmittance in the visible spectral range from 400 nm to 635 nm. A detailed description of this SRM is given in NBS Special Publication 260–51 [2]. SRM 931 is a liquid standard which consists of a solution of cobalt and nickel in dilute perchloric acid contained in glass ampoules [3]. The transmittance of these solutions is certified from 302 nm to 678 nm and should be used in conjunction with curvettes having a known light path; such curvettes are available from NBS as SRM 932 [4].

Both SRM’s 930 (the glass filters) and 931 (the liquids) are limited in their spectral transmittance range and require the use of spectral bandpasses, from 2.2 nm to 6.5 nm for the glass filters and from 1.0 nm to 6.5 nm for the liquid filters depending of wavelength, when accurate transmittance values are sought.

The need to provide similar transfer standards, but with expanded spectral range to the ultraviolet and with less stringent spectral bandpass requirements, has resulted in an investigation to find adequate materials for this purpose. Such materials should fulfill the following conditions: (a) be transparent in the spectral range of interest, usually between 200 nm and 800 nm; (b) have a transmittance independent of wavelength (optically neutral); (c) have a spectral transmittance independent of temperature; (d) have low reflectance and be free of interferences; (e) be nonfluorescent; (f) be stable, homogeneous, and free of strain; (g) have mechanical stability for the size used (thickness, length, width) and be easy to fabricate by conventional techniques used in optical shops; (h) be simple to use in conjunction with the conventional spectrophotometers available today in analytical laboratories; (i) be readily available and relatively inexpensive. Thus, various solid materials were examined and the final choice was the evaporated metal-on-(non-fluorescent) fused silica type filter.

The transmission characteristics of such filters are illustrated in figure 1 and are compared with that of three glass filters and Ronchi ruling on glass.

The major limitation of the evaporated metal-on- quartz filters results from their intrinsic property of attenuating the incident radiation by reflecting rather than absorbing part of it. As a consequence of this property, this type of filter could generate stray radiations in the sample compartment of conventional spectrophotometers and is susceptible to produce interreflections when used with instruments equipped with lenses.

To determine the practical value of such filters as a transfer transmittance standard, comparative measurements were performed with the National Physical Laboratory in England (NPL), and the results are given in table I. In this case the inconel-on-fused silica filter was protected by a clean fused silica plate held in place with an organic cement. Except for a filter which showed some mechanical flaws in its structure, the reproducibility of transmittance measurements was as good as that obtained for the absorbing glass filters (table II). The error which would result from positioning the inconel-on-fused silica was measured by rotating from 0° angle to 3°—6°—and 9°. From the results obtained (table III) it can be concluded that in the case of the high- accuracy spectrophotometer used at NBS a positioning error within 3° can be tolerated.

The interreflection error affecting the measurements when a filter is inserted in the radiation path between the two lenses of a spectrophotometer was examined in detail [5]. The measured error for a glass filter established for the high-accuracy spectrophotometer was one or two 10^−4^ transmittance units; a value which is about four times larger was found for an evaporated metal-on-fused silica filter. It should be mentioned here that both the positioning and interreflection errors are, for a given material, instrument dependent.

As a result of these considerations a decision was made to produce a limited number of sets of evaporated inconel-on-fused silica and to test these filters in actual measurements performed on the conventional spectrophotometers manufactured in the United States.

One of the filters from a set is shown in figure 2. From left to right one can see the main body of the filter holder made from an aluminum alloy anodized black. This body is provided with dove-tail grooves which can accept a front and rear shutter made from a black plastic (Delrin^2^). The last three units illustrate the holder with these shutters on. A more detailed dimensional drawing of the filter holder is shown in figure 3. The shutters provide two functions: one is to protect the filter from contamination, the other to detect if stray radiant energy (SRE) is produced in the spectrophotometer compartment as a result of reflections generated by the incident beam on the semi-transparent metal layer. The determination of stray radiant energy generated in the sample compartment of the spectrophotometer, due to the reflecting propreties of these filters, may be assessed as follows: A background signal may be measurable when the instrument shutter at the photomultiplier tube is closed. Another slightly higher background signal may be detectable with the instrument shutter open and with the filter placed in the beam, in the sample compartment, with both sliding shutters closed. Both of these signals should be very small. A third signal may be detected when the front sliding shutter at the filter holder is removed and the rear sliding shutter is closed. If under these circumstances a signal is detected, it is very likely due to SRE produced by reflections resulting from the semi-transparent mirror which scatter from the walls of the cell compartment. This should also be low in magnitude. The transmittance of the filter is measured where both sliding shutters at the filter holder are removed.

Under these circumstances a source of systematic error can be from multiple reflections between the lenses in the instrument and the filter surfaces [5]. An indication of the existence and magnitude of such interreflection phenomena can be obtained by comparing the differences between the transmittance measurements and the certified values for SRM 930 and 931 to the corresponding differences for the inconel-on-fused silica filters.

Each set is made from three filters and one blank placed in individual metal holders which are provided with the front and rear sliding shutters. The selection of inconel as the semi-transparent metal layer was made on the basis of its relatively good optical neutrality in the spectral range from 250 nm to 700 nm. The fused silica substrate is of optical quality and non-fluorescent. All the silica filters, including the blank, were ground and polished at the same time and together to a parallelism of 0.02 mm and a flatness of less than 2 fringes (mercury 546.1 nm). The nominal dimensions are 30.5 mm by 10.4 mm by 2 mm thick, and the nominal transmittances of these filters are 1 percent; 20 percent; 30 percent; and 90 percent.^3^ The transmittances of each set were measured at 250 nm, 300 nm, 340 nm, 400 nm, 440 nm, 465 nm, 500 nm, 546.1 nm, 590 nm, and 635 nm. Sets of these filters were sent to the manufacturers of spectrophotometers for evaluation and tests on the individual instruments. The results of these tests will permit to establish the usefulness of these filters as SRM’s in spectrophotometry.

In the mean time comparative measurements were performed on two conventional spectrophotometers available at NBS, and the results are assembled in table IV. These results are preliminary measurements and should be considered with caution. The data seem to indicate, however, that the evaporated metal-on-fused silica filters may be an acceptable material for use as transfer standards in spectrophotometry.

## Figures and Tables

**Figure 1:**
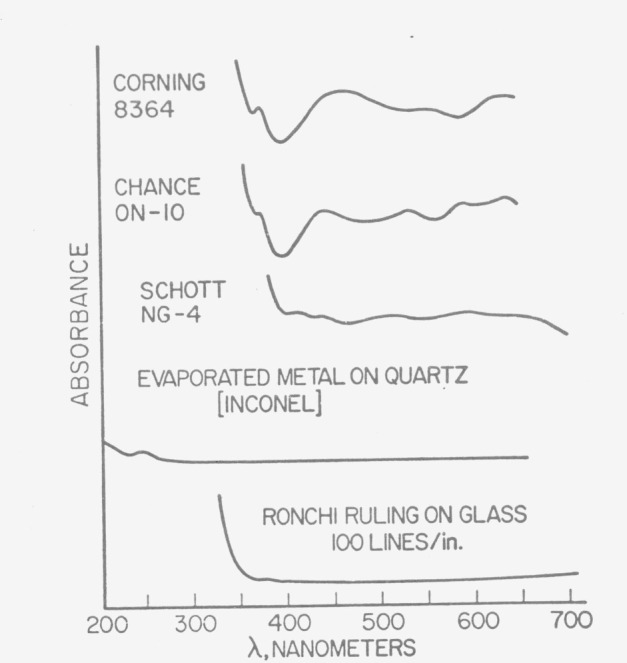
Spectral characteristics for five transparent materials from 200 nm to 700 nm.

**Figure 2:**
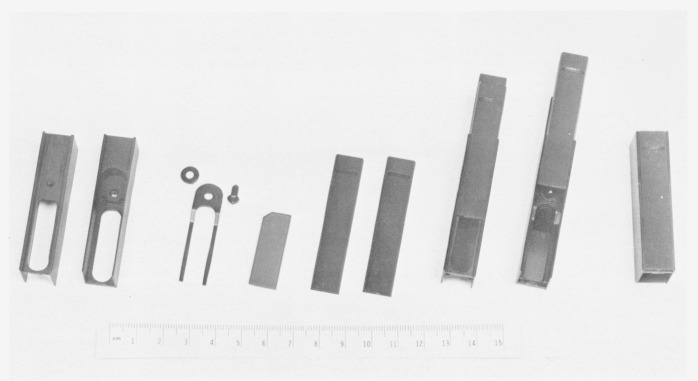
Filter holder with shutters. From left to right: front and rear view of the holder body provided with dove-tail grooves; retaining spring with nylon screw and washer; filter; two shutters; front view of the filter holder with front shutter; rear view of the filter with rear shutter; filter holder with both shutters closed.

**Figure 3:**
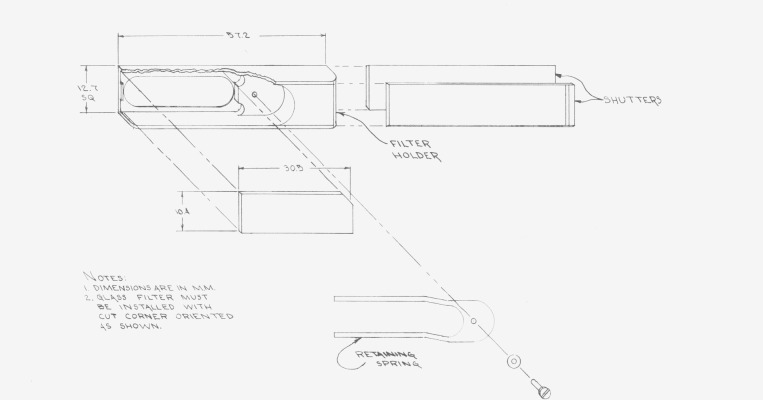
Details of the filter holder with shutters. Nominal dimensions are in millimeters.

**Table I:** Comparison between the percent transmittances (%T) measured on three inconel-on-silica filters at NPL and NBS

Wavelength nm	NBS, *%T*		NBS, *%T* average	NPL, *%T*
	1	2		
				
450.0	24.87	24.88	24.87_5_	24.93
550.0	23.78	23.82	23.80	23.86
650.0	23.38	23.39	23.38_5_	23.46
450.0	49.35	49.33	49.34	a49.56
550.0	47.60	47.60	47.60	47.81
650.0	46.85	46.85	46.85	47.14
450.0	72.17	72.20	72.18_5_	72.30
550.0	72.05	72.11	72.08	72.20
650.0	72.20	72.34	72.27	72.33

Average difference between NBS and NPL percent *T* values = −0.30 percent

a This filter had a flaw in the form of a crack which was sometimes visible and other times invisible. The larger differences found in the measurements of this filter may be due to this flaw.

**Table II:** Comparison between the percent transmittances (%T) measured on three Schott NG-4 glass filters at NPL and NBS

Wavelength nm	NBS, *%T* March 12, 1971	NBS, *%T* May 18, 1971	NBS, *%T* average	NPL, *%T* February 1971
				
440.0	12.92	12.91	12.91_5_	12.93
465.0	14.96_5_	14.98	14.97_3_	15.01
590.0	11.70	11.64	11.67	11.67
635.0	12.72	12.68	12.70	12.72
440.0	19.62_5_	19.58	19.60_3_	19.62
465.0	22.38_5_	22.35	22.36_7_	22.43
590.0	19.06	18.95	19.00_5_	19.01
635.0	20.45_5_	20.37	20.41_3_	20.47
440.0	32.89	32.86	32.87_5_	32.98
465.0	35.52	35.54	35.53	35.66
590.0	31.16_5_	31.10	31.13_3_	31.21
635.0	32.56_5_	32.52	32.54_3_	32.62

Average difference between NBS and NPL percent *T* values = −0.19 percent

**Table III:** Percent transmittance (%T) measured on a Schott neutral glass filter 2 mm thick, and an inconel-on-fused silica filter 2 mm thick at 590 nm, for normal incidence and for the angle of 3°; 6°; and 9°

%T			
Filter	Schott NG-4 Glass	Inconel-on-fused silica	
Angle		Front	Back
			
0°	28.13	29.91	29.87
3°	28.10	29.98	29.87
6°	28.03	29.84	29.82
9°	27.98	29.92	29.90

**Table IV:** Percent transmittance (%T) measured on four inconel-on-fused silica filters at 10 wavelengths and on the NBS-IMR high-accuracy spectrophotometer and on two conventional instruments A and B

*%T*										
Wavelength nm	250	300	340	400	440	465	500	546	590	635
Instrument										
										
NBS-IMR	1.45	1.81	1.86	1.94	2.04	2.13	2.27	2.49	2.70	2.90
High-accuracy Spectrophotometer	21.39	21.27	20.33	18.93	18.33	18.09	17.90	17.83	17.86	17.95
	28.68	29.92	30.20	29.96	29.70	29.55	29.44	29.44	29.49	29.57
	91.42	92.34	92.66	92.90	93.01	93.05	93.11	93.16	93.19	93.23
Spectrophotometer A	1.53	1.84	1.93	2.00	2.12	2.20	2.34	2.55	2.75	2.95
	21.37	21.24	20.31	18.91	18.29	18.07	17.89	17.82	17.84	17.92
	28.67	29.87	30.19	29.95	29.70	29.58	29.49	29.46	29.52	29.60
	91.59	92.60	93.32	93.20	93.30	93.30	93.42	93.46	93.50	93.58
Spectrophotometer B	1.49	1.85	1.90	1.97	2.07	2.16	2.31	2.53	2.83	3.06
	21.6	21.5	20.5	19.1	18.5	18.3	18.1	18.0	18.3	18.5
	28.8	30.0	30.2	30.0	29.9	29.7	29.5	29.5	29.9	30.0
	91.4	92.6	92.6	93.1	93.3	93.3	93.3	93.3	93.9	93.9

## Footnotes

1 Figures in brackets indicate the literature references at the end of this paper.

2 In order to describe materials and experimental procedures adequately, it was occasionally necessary to identify commercial products by manufacturer’s name or label. In no instance does such identification imply endorsement by the National Bureau of Standards, nor does it imply that the particular product or equipment is necessarily the best available for that purpose.

3 The filter holders and the evaporated inconel-on-fused silica filters were made in the NBS Instrument Shops.
